# Tea, talk and technology: patient and public involvement to improve connected health ‘wearables’ research in dementia

**DOI:** 10.1186/s40900-017-0063-1

**Published:** 2017-08-01

**Authors:** Lamiece Hassan, Caroline Swarbrick, Caroline Sanders, Angela Parker, Matt Machin, Mary P. Tully, John Ainsworth

**Affiliations:** 1 Farr Institute of Health Informatics Research, School of Health Sciences, Faculty for Biology, Medicine and Health, The University of Manchester, Oxford Road, Manchester, M13 9PL UK; 2 NIHR School for Primary Care Research, The University of Manchester, Oxford Road, Manchester, M13 9PL UK; 3 NIHR CRN Greater Manchester, Central Manchester University Hospitals NHS Foundation Trust, North Road, Manchester, M13 9WL UK; 4 School of Health Sciences, Faculty for Biology, Medicine and Health and The Manchester Pharmacy School, The University of Manchester, Oxford Road, Manchester, M13 9PL UK

**Keywords:** PPI, Patient and public involvement, Public engagement, Dementia, m-health, Health informatics, e-health, Wearable, Physical activity

## Abstract

**Plain English summary:**

There are a growing number of mobile phones, watches and electronic devices which can be worn on the body to track aspects of health and well-being, such as daily steps, sleep and exercise. Dementia researchers think that these devices could potentially be used as part of future research projects, for example to help spot changes in daily activity that may signal the early symptoms of dementia. We asked a range of older people, including people living with dementia and their carers, to participate in interactive discussions about how future participants might find using these devices as part of research projects. We also invited volunteers to borrow a range of devices to test at home, giving them further insights. Discussions revealed that people were generally supportive of this type of research, provided they gave informed consent and that devices were discreet, comfortable and easy to use. They also valued technical support and regular feedback on study progress to encourage ongoing participation. These findings were used to develop a pool of devices for researchers, with computer software and written guidance to help plan, design and support studies. Our work shows that when given the right opportunities, people who are affected by dementia can provide valuable insights that can enhance the design, delivery and quality of future research.

**Abstract:**

**Background**

Increasingly, researchers are recognising the potential for connected health devices, including smartphones and smartwatches, to generate high resolution data about patterns of daily activity and health outcomes. One aim of the Dementias Platform UK (DPUK) project is to provide researchers with a secure means to collect, collate and link data generated by such devices, thereby accelerating this type of research in the field of dementia. We aimed to involve members of the public in discussions about the acceptability and feasibility of different devices and research designs to inform the development of a device pool, software platform and written guidance to support future studies.

**Methods**

Over 30 people attended a series of interactive workshops, drop-in sessions and meetings in Greater Manchester. This included people living with dementia and cognitive impairments, carers and people without memory problems. Discussions were tailored to suit different audiences and focused on the feasibility and acceptability of a range of different wearable devices and research designs. We also invited volunteers to borrow a device to test at home, enabling further insights from hands-on interactions with devices.

**Results**

Discussions revealed that people were supportive of connected health dementia research in principle, provided they gave informed consent and that devices were discreet, comfortable and easy to use. Moreover, they recommended technical support and regular feedback on study progress to encourage ongoing participation.

**Conclusion**

By using a range of discussion-based and practical activities, we found it was feasible to involve people affected by dementia and use their insights to shape the development of a software platform and device pool to support future connected health dementia research. We recommend that researchers planning such studies in future pay adequate attention to designing suitable participant information, technical support and mechanisms of providing study progress updates to support sustained engagement from participants.

## Background

Dementia is an umbrella term for a range of neurodegenerative diseases, causing progressive damage to the neurons of the brain resulting in a deterioration of cognitive function. People living with dementia can experience problems with memory loss, cognitive abilities, language and communication. In the UK it is estimated that there are over 850,000 people living with dementia, 40,000 of whom are aged under 65, known as young onset dementia [1]. If the current prevalence rate remains stable, this figure is projected to exceed 1 million by 2025 in line with the ageing population [1].

Whilst the cost of dementia to the UK economy has been estimated at almost £26 billion per year [1], spending on dementia research in the UK has been disproportionately low [2]. More recently, however, dementia has been identified as an international research priority, with funding in the UK set to double over the next decade [2]. Contributing to this global effort, the Medical Research Council established the Dementias Platform UK (DPUK) in June 2014, which represents a multi-million pound investment linking academia and industry to accelerate dementia research [3]. The hope is that this will enable advanced tools, techniques and analysis that will lead to new insights into the detection, prediction, treatment and understanding of the disease.

Health informatics, the field of study concerned with the management and use of information in healthcare [4], is central to the vision of DPUK. One emerging area of work is to provide researchers with secure means to collect, collate and link data generated by newer connected health devices, in particular smartphones and wearable activity trackers (henceforth ‘wearables’), worn on or close to the body. An increasing number of commercially available devices now use combinations of sensors, algorithms and applications to offer digital tracking functions across various measures of health and well-being, such as physical activity, sleep duration and heart rate. Whilst most are primarily marketed as tools to achieve behaviour change goals (e.g. weight loss or fitness), health researchers across a variety of fields are increasingly recognising the potential for such devices to capture high-resolution, multi-dimensional data from everyday life [5–7].

With respect to dementia, several hypotheses have emerged which link changes in cognitive function with sleep, obesity and physical activity [8, 9]. Thus, it is possible that future studies might use wearables in both an observational and an interventional sense. Longitudinally, wearables could be used to passively and unobtrusively monitor patients’ progress and generate relevant data to act as predictors or clinical endpoints in research studies, for example signals and predictors associated with decline in cognitive and physical functioning. Another angle may be to use certain devices as health interventions in themselves; for example they might use persuasive feedback techniques (e.g. gamification) to promote increases in physical activity, or act as monitoring systems to track user locations with the aim of supporting patient safety.

DPUK committed to providing a combination of hardware and software to encourage research using connected health devices: a pool of devices (available for loan) capable of generating data and a supporting ‘sensing platform’ designed to securely receive and store these data. Data stored within the sensing platform may be analysed by itself or linked with other data sources, for example clinical records detailing specific diagnoses, treatments and clinical outcomes over time.

To gain maximum value from the sensing platform, the approaches and devices to be used as part of future research projects need to be acceptable to prospective participants, as well as suitable for scientific purposes. The success of projects may, for example, be affected by perceptions relating to relative ease of use, features, comfort, privacy and security offered by different devices used for research purposes. Involving people with dementia or cognitive impairments in influencing future research is not without challenges; for example, in addition to memory problems, people with dementia may experience difficulties with visual perception and understanding complex information. Therefore to support people to be involved, adequate attention needs to be paid to choosing easily accessible venues for activities, providing straightforward written and verbal information and considering the pace and structure of activities [10]. Nonetheless, involvement is justifiable on both moral and quality grounds. Involvement may increase the relevance of future research to patients and the wider public, as well as optimising methodological aspects, for example enhancing recruitment and retention strategies [2, 11].

In this paper, we describe how we involved members of the public to (i) inform design and procurement decisions regarding the sensing platform and device pool and (ii) produce general guidance to optimise study design and improve acceptability for those planning and conducting dementia research using mobile connected health devices.

## Methods

During the period of October 2015 to January 2016, over 30 members of the public - including people living with dementia, carers and people with and without memory problems - attended workshops, drop-in sessions and meetings designed to elicit feedback on the acceptability of a range of wearable devices for capturing data for different study purposes and to produce guidance about their usage in future research studies.

The people involved in these activities were acting as advisers about the design of future research, often based on their experience of living with dementia and/or acting as carers. Therefore, these activities constituted involvement, rather than research participation and, in line with current guidance from the National Research Ethics Service and INVOLVE [12] ethical approval was not required. We sought and gained confirmation from The University of Manchester’s Research Ethics Committee that this was indeed the case before proceeding. It was made clear to workshop attendees that we were interested in their views of how the subjects under discussion related to other people in similar circumstances who might be recruited to future research studies, rather than only their personal views. Nonetheless, we did consider ethical issues when conducting our activities.

### Pre-meeting for researchers

**Fig. 1:**
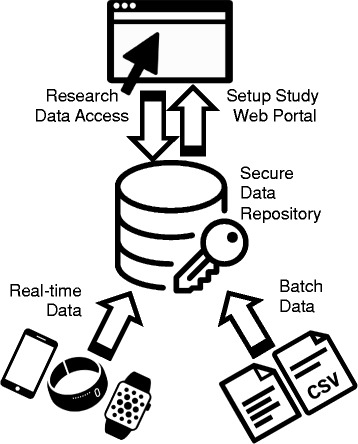
Architecture of the DPUK sensing platform . Legend/Description: Registered users can setup their research study and add data using a secure web portal. Details can be added about study participants and data collection encounters. Real-time data can be uploaded from a range of devices in a variety of formats. The architecture of the platform is flexible to allow for new devices to be linked in the future. Batch data from third parties (e.g. hospital records data) can be linked with device data for analysis purposes. All data are uploaded to a secure data repository located at The University of Manchester

On 6th October 2015, a half-day meeting was arranged with researchers, the primary users of the DPUK software platform, to understand how they wanted to use the sensing platform and allow them to influence the platform’s initial focus and outcomes. The meeting was hosted by the developers of the platform and attended by academics and clinicians from the dementia research community (public and private sector). Following an overview of progress, researchers were invited to comment on the platform, which was designed to be capable of receiving and storing data from a range of wearable devices (see Fig. 1). Interactive exercises were used to scope the range of potential future studies where the platform might be used and to identify the technical requirements for devices to be connected to the platform. As a result, an initial range of wearable devices to appraise were identified alongside a series of possible research questions and likely methodologies, providing context to their use.

### Devices purchased for testing

Following the researchers’ workshop, we purchased a selection of devices in various colours and sizes (see Table 1) for further testing and use within patient and public involvement activities. Prior to activities, researchers set up and wore devices to generate exemplar data and to familiarise themselves with device set-up procedures, functions and interfaces. Devices were synchronised with various smartphones and tablets (both Android and Apple).

**Table 1:** Features of wearable activity devices tested

Device	Steps	Sleep	Heart rate	Waterproof	Display	Mounts	Retail price (approx.)	Battery life
Axivity AX3	N/A^a^	N/A^a^	X	✓	None	Wrist, belt	£99	30 days
Fitbit Charge HR	✓	✓	✓ (continuous)	X	Digital	Wrist	£120	5 days
MOTO 360	✓		✓ (on demand)	X	Digital	Wrist	£175	24 h
Garmin Vívofit 2	✓	✓	✓ (on demand)	✓	Digital	Wrist	£90	1 year
Misfit Speedo Shine	✓	✓	X	✓	Dial	Wrist, magnetic clasp	£60	6 months
Withings Activité Pop	✓	✓	X	✓	Analogue	Wrist	£120	8 months
Withings Pulse Ox	✓	✓	✓ (on demand)	X	Digital	Wrist, belt	£70	2 weeks

^a^Outputs are raw, unprocessed data, so require further analysis to convert into discernible states and activities

### Deciding who to involve

We wanted to involve people similar to those who could potentially be asked to wear devices as part of future research projects. We identified four distinct patient and public groups to focus on:

1. People living with dementia and their carers.
2. People living with memory problems or Mild Cognitive Impairment (MCI) and their carers.
3. People living with dementia aged 65 years or younger, referred to as young onset dementia.
4. People without known memory problems aged 50 years and older.

To optimise content and delivery style, separate activities were planned for each group. Table 2 provides an overview of attendees, activity formats used and advertising methods. A combination of posters and written materials were designed to advertise involvement opportunities and circulated via physical and electronic (i.e. social media and email) formats. Refreshments and payments (£20 per session) were offered to acknowledge the valuable contributions of individuals, as is regarded as good practice [13]. For pragmatic reasons, events were all held within Greater Manchester.

**Table 2:** Description of patient and public attendees, by group

	Group			
	Living with dementia	Memory problems and MCI	Dementia < 65 yrs	No known memory problems > 50 yrs.
Number	5 + 4 carers.	2 + 1 partner.	Approx. 8–12 (inc. some carers)^a^.	9.
Gender	5 women, 4 men.	1 woman (and partner), 1 man.	Men and women.	5 women, 4 men.
Age group	All but one aged >65 years.	All >65 years.	All <65 years.	Mainly mix of 50–60 and 60–70, at least 1 aged 70 + .
Activity format	Workshop at a Salford-based dementia resource centre	1:1 meetings	Activity at a Salford-based weekly drop-in support group meeting	Workshop at The University of Manchester
Invited via	Age UK, a Salford-based dementia resource centre and Open Doors Network.	Ongoing research study recruiting people with memory problems.	Attending a Salford-based weekly support group.	University PPI networks, University admin/support staff, social media.
Number of devices loaned	4 - Fitbit Charge HR, Misfit Speedo Shine and Withings Activité Pop.	2 - Fitbit Charge HR and Garmin Vívofit 2.	0 - one-off activity.	6 - Fitbit Charge HR, MOTO 360, Garmin Vívofit 2, Misfit Speedo Shine, Withings Activité Pop and Withings Pulse Ox.

^a^This was a drop in activity, which took place within an open space. Therefore, we can only provide approximations for the numbers and types of people who took part

### Workshops and meetings

Workshops were planned, consisting of two meetings over the course of one to two weeks, giving people the option to test the devices at home in between sessions. Sessions were designed to be stimulating and interactive, allowing people the opportunity to view, handle and try on a range of devices. Sessions covered the following content:

- *Session 1 -* Introduced types of dementia research and questions, introduced devices, discussed using devices for research, gave opportunity to handle devices and test functions, invited volunteers to borrow a device to test at home.
- *Device testing period (1 week) –* Volunteers invited to use a device, view data and reflect on ease of use, wearability and barriers to use. Twelve individuals/couples took up this opportunity (see Table 1).
- *Session 2 -* Shared and discussed experiences of using devices at home, considered which devices might be most suitable for different research participants in future, reflected on user support requirements, data governance and privacy issues.

A series of supporting guides and documents were developed to support the workshops, including discussion guides, example research scenarios (see Table 3) and printed summaries of specifications for individual devices.

**Table 3:** Research scenarios

Ideas from the researchers’ meeting were used to generate a series of hypothetical, but plausible, scenarios where dementia research might use connected health technology. These were used in patient and public workshops to provide context to the use of wearable devices in dementia research and to prompt further discussion. Scenarios included observational and interventional research designs. Below is an example of an observational research scenario that was adapted for use in different groups.
*Example scenario: The cohort study.*
“Participants taking part in a birth cohort study (a long-term study where people are tracked throughout their lives) are asked to take part in a spin-off study to see if staying physically active has any impact on their mental abilities and likelihood of developing dementia.
Throughout their lives, participants have already completed questionnaires about their lifestyle habits and have submitted biological data (e.g. blood tests, saliva) to the study. Details about their health conditions and care have also been collected from their medical records, with their permission. For this study, they are asked to wear an activity tracker for one month every year to measure their usual steps, distance and heart rate. The data generated from the trackers are then matched with participants’ other data from the study and their medical records to see if there is any association between levels of physical activity and changes in mental abilities, such as memory, reasoning, language and attention.”

Two-session workshops were subsequently delivered for two groups: people living with dementia and their carers, and people without known memory problems. In the case of people living with MCI or memory problems and their carers, we did not attract a sufficient number of people to make it feasible to run a workshop. In this case, we met with people individually, covering similar content to that delivered in the workshops, albeit in a more informal manner. To gain input from people living with young onset dementia, we were invited to attend a Salford-based support group (facilitated by Age UK, Salford) and spoke with small groups in turn at one of their weekly meetings. Workshops were facilitated by researchers experienced in public involvement and working with people living with dementia. Activities were designed to be interactive, visual and delivered at an appropriate pace for attendees to enable their participation.

### Device testing

Individuals were offered the opportunity to borrow a device of their choice (subject to availability). This was purely to allow individuals the opportunity to judge usability and thus make recommendations from a position of greater insight: we made no attempt to collect or analyse the data generated by the borrowed devices. We took a number of steps in order to respect autonomy and privacy of individuals. Firstly, individuals were offered a device of their choice (subject to availability) to test on a purely voluntary basis and they were invited to attend the second workshop regardless of their decision. Information about each of the devices was provided and signposted (e.g. hard copy of instructions provided in the box, user terms and conditions). We also summarised and explained key points, including what data would be gathered by the device and who they would be shared with (e.g. the device manufacturer). Following testing, any data held on devices borrowed were deleted (using a factory reset). We also explained to individuals how to delete their individual user accounts if they wished and offered technical support where necessary.

### Development and implementation of recommendations

At the workshops, detailed notes were taken by additional assistants (researchers and software developers). Attendees were also encouraged to record ideas on flipcharts and post-it notes. Some also spontaneously submitted their own written reports or emailed feedback following activities. After each workshop or activity, facilitators collated information from personal notes, flipchart notes, photographs and evaluation forms into individual reports, which stated key findings. Some direct quotes were also used within these reports, which originated from comments written on post-it notes or reports. Reports were shared with workshop attendees to check for accuracy of reporting and interpretation. In turn, they were then disseminated and discussed among the wider project team. In January 2016, a summary report of all activities was produced, complete with recommendations for purchasing devices, software infrastructure and the conduct of future research. The summary report was also disseminated to workshop attendees for comment and subsequently made available online for researchers. The lead author (LH) was then invited to subsequent meetings regarding purchasing decisions to provide input into decision-making in line with the recommendations and to record which recommendations were subsequently implemented.

## Results

This section is divided into five main themes, each discussed in turn: benefits of participating in dementia research; device preferences; data security, usage and storage; technology set-up and support; and retention and engagement. In addition, the recommendations that were developed as a result and, where possible, how they were acted upon are also presented. The main feedback points and recommendations for each theme are summarised in Table 4.

**Table 4:** Summary of theme, feedback and recommendations

Theme	Feedback	Recommendation
Benefits of participating in connected health dementia research	• People were interested in how connected health research could benefit prospective participants and/or others.	• Information provided to prospective participants should include a) prospective benefits to individual participants and future patients and b) information on what would happen if researchers picked up something irregular, indicating a potential health problem.
	• People expected researchers to intervene if devices picked up clear signs of treatable health problems requiring medical attention.	
Device wearability	• Individuals varied in terms of which devices they found most tolerable, useful and aesthetically pleasing to wear.	• More than one type of device should be purchased, with a variety of straps and mounts in different colours, sizes and materials*.
	• People preferred devices that were low maintenance and robust.	• Devices to be worn for longer periods should be waterproof and should have a long-life battery*.
	• Some concerns that devices might draw unwanted attention or reveal an individual’s health status.	• Devices should be unobtrusive, either passing for a wristwatch or be capable of being worn discreetly elsewhere*.
Data security, usage and storage	• People generally trusted University researchers to protect personal and device data and treat it confidentiality.	• Software and devices used (inc. any associated 3rd party software applications) should provide the necessary security to protect personal data and ensure privacy*.
	• People accepted that using certain devices for research might entail sharing data with commercial companies, but expected to be told upfront.	• Information provided to prospective participants should include information on how data is transferred between the device, researchers and any third parties.
Technology set-up and support	• Few of those living with dementia and their carers had access to smartphones and/or tablets of their own.	• A stock of phones and/or tablets should be purchased to enable people who do not own their own suitable device to participate*.
	• Some people required a high level of support to set up and use devices and applications running on smartphones and/or tablets.	• Set up of devices should include an offer of in-person technical support to assist with device set up, check-ups, maintenance and troubleshooting.
Retention and engagement	• Feedback on progress and outcomes could be an incentive for wearing devices for longer, making people feel valued and engaged.	• Researchers should offer participants feedback on study progress and outcomes throughout the study in a choice of formats.
	• Some people who acted as ‘testers‘ were unable to tolerate certain devices, whilst others unexpectedly enjoyed wearing them.	• Researchers should consider offering a trial period in advance of the research, without obligation to participate in the study.
	• Carers were deeply involved in the everyday routines of people living with dementia.	• Researchers should offer to involve carers throughout the process of recruitment, follow ups and dissemination.

^*^indicates recommendation has been implemented

### Benefits of participating in dementia research

We sought general feedback on the premise of using connected health devices to advance dementia research, using a series of hypothetical exemplar research scenarios. Patients and members of the public across all groups were very supportive in principle of the purpose of this type of research. Indeed, all of the people we spoke to agreed that they would be happy to wear a device for at least some period of time as part of dementia research, provided they gave informed consent to do so.

Discussions about willingness to participate in dementia research often gravitated towards the prospective benefits; to individual participants, to people living with dementia or cognitive impairments, and to society. Whilst not viewed as essential, people were interested in whether wearing devices as part of research could benefit prospective participants personally.

Several people without memory problems mentioned an interest in using devices and feedback for health and fitness purposes; those living with dementia were more concerned with potential improvements in quality of life, although these were not mutually exclusive. Furthermore, a few individuals also asked about the potential for devices to act as an ‘early warning surveillance system’ for potential health problems. This, in particular, raised discussions around the potential ethical issue as to what would happen if researchers picked up an irregularity, indicating a potential health problem either related to dementia (e.g. cognitive impairment) or unrelated (e.g. very high heart rate). Under such circumstances, there was an expectation that researchers should intervene if there were clear signs of treatable health problems requiring medical attention.

Devices which enabled sleep tracking as well as physical activity were particularly popular, although for subtly different reasons. Some individuals were merely interested in tracking sleep out of general interest i.e. how well do I sleep? Meanwhile, others were more concerned with understanding sleep patterns for self-management purposes. In particular, those living with dementia and their carers spoke of inadequate sleep, interrupted sleep, frequent awakening and sleeping at inappropriate times.

As a result of this feedback, we recommended that information provided to prospective participants of future dementia research should include information on a) any immediate prospective benefits to individual participants and any future benefits to patients and b) how researchers would act in the event of suspecting a treatable health problem.

### Device wearability

We gave people the opportunity to view, handle and try on a range of devices at workshops and meetings. Some also took the opportunity to borrow a device to take home to test. The devices we used came in various different colours, sizes and materials. All could be worn on the wrist, although some had different mounts allowing them to be worn elsewhere on the body (see Table 1).

Individuals varied in terms of how they preferred to wear devices and which they found most wearable, useful, usable and aesthetically pleasing. In general, however, people preferred devices that were low maintenance, robust and provided some form of personal feedback, especially if displayed on the wrist. Resistance to water was seen as important across all groups:

*“It was a nuisance to have to take it off and store it when, for example, taking a shower after working out at the gym.”*

Some people proposed different requirements for wearing a device for research purposes than for general personal use. Even if an individual had no interest in the feedback themselves, they might still be willing to wear a device in the name of research. One person in particular distinguished between whether the research required participants to wear and use the device *actively* (e.g. as a tool to self-manage physical activity) or *passively;* as he described it, “*wear it and forget about it*.”

A few individuals – particularly those with dementia, memory problems and/or MCI - worried that wearing a device might draw unwanted attention from others that could lead to the person’s health status being questioned or revealed. One of the most popular devices resembled an analogue watch, which was especially welcomed for long-term use: “*I’d be happy to replace my watch with this*”.

As a result of this feedback, a selection of devices were ultimately purchased that met the requirements in Table 4 and were capable of supporting a range of different research projects. Furthermore, the architecture of the data storage platform was developed to be ‘device-agnostic’, allowing for new devices to be connected in future. We also supplemented devices with a variety of additional straps and mounts in different colours, sizes and materials (including leather and synthetic). All devices were waterproof and all except one had a long-life battery of 6 months or more. Devices were also unobtrusive enough to either a) pass for a wristwatch or b) be worn discreetly elsewhere, therefore not inviting unwanted questions. One notable exception among devices purchased was the Axivity AX3. This particular device did not display the time; however, it provided detailed 3-axis accelerometer data in an open source format, which would allow the research team to perform their own analysis on the raw data.

### Data security, usage and storage

Questions were asked about how researchers would access and use data generated by devices. People generally trusted University researchers to handle device data, provided reassurances were given that data would be treated confidentiality, anonymised where possible and stored securely. In any case, the people we spoke to perceived the data being collected as predominantly low risk and not especially sensitive. As one person questioned, “*how could anyone use it*”? One important exception to this was where studies proposed using more detailed location (GPS) data, which individuals stated would warrant more careful justification and explanation.

Facilitators explained that in most cases, using ‘off the shelf’ devices meant that any data generated would be sent to the commercial companies who had developed the product and associated software, who would then be asked to release the data to the research team. The people we spoke to were willing to accept these arrangements. Some acknowledged that anyone who bought a device independently, rather than as part of a research project, would have to accept sharing their data with commercial companies in any case. One device that allowed participants to send their data directly to the research team was displayed and discussed (the Axivity AX3). However, this particular model did not include a display or enable personal feedback and was not well-received as an alternative to the other devices seen. Under such circumstances, the proposed data sharing arrangements were seen as acceptable, providing participants were informed of these in advance of their participation.

As a result of this feedback, we recommended that information should be provided to prospective participants of future research as to how data are transferred between devices, researchers and any third parties. In line with this feedback, the sensing platform has been designed to provide the necessary security to protect personal data and ensure privacy. The DPUK sensing platform is hosted within a secure infrastructure at the Farr Institute and complies with best practices on data security and information governance. All data transfer to and from the sensing platform is conducted over secure encrypted channels.

### Technology set-up and support

We encountered wide variation among people in terms of their experience with technology, computing skills and ownership of smartphones and/or tablets. The set up process typically involved: preparing the device for use (e.g. inserting the battery); installing an application on a smartphone and/or tablet; providing personal details (e.g. email address); and pairing the device with the smartphone/tablet. Many of those who owned their own devices chose to set these up themselves at home and generally accomplished this successfully, with few problems. This was most common among people without known memory problems. However, some people required much more support, especially those who did not routinely use smartphones, tablets and/or computers. This was based both on self-report during discussions and directly observed where individuals volunteered to borrow a device to trial at home.

Once set up, we found that people living with dementia and their carers also required more help to navigate device settings and interpret the data. Few of the people in this group that we spoke to had smartphones or tablets of their own and none had used wearable devices previously. Some particularly struggled when viewing and interacting with data provided via the application (rather than on the device itself). This appeared to be partly down to the complexities of various interfaces, limitations in manual dexterity as well as screen size. Indeed, many of the people we spoke to, across all groups, preferred to use tablets rather than smartphones to use applications and view data. As a result of this insight, we purchased a stock of smartphones and tablets as part of the device pool, specifically to enable people who do not already own a suitable device to participate in future research projects.

Whilst people living with dementia and their carers did seem to require more support to set up and use applications, abilities varied from person to person: some individuals were highly competent users whilst others had little familiarity with technology. Regardless of their individual abilities, several of the people we spoke to specifically warned against the dangers of stereotyping and urged researchers to treat people as individuals when considering support needs, rather than making assumptions based on age or health status. As one carer commented:

*“You can’t say that everyone over the age of 60 can’t cope with technology, it just isn’t true.”*

Therefore, we recommended that researchers should offer in-person support to all prospective participants for set-up of devices. Furthermore, researchers should consider building in appropriate provision for technical support (preferably via both telephone and email), to support ongoing participation and troubleshooting.

### Participant retention and engagement

Most of the research scenarios described adopted a longitudinal design, thereby requiring participants to wear devices for a period of weeks, months or even years. A number of potential suggestions to improve retention were also identified by patients and members of the public.

People expected that participants would be very interested in the findings of research. Many thought that providing personalised feedback to participants, regular updates on progress and outcomes throughout the research (not just at the end) could be an incentive for wearing devices for longer, making people feel valued and engaged. Some individuals had previously taken part in research, but were not always made aware of the results. One couple said that they had been invited to a talk about results from a project, but this had been too far to travel and no other options for feedback were available: “*we made all that effort and we never even found out what happened*”.

Even though studies in this area may be focused around electronic applications and devices, it did not necessarily follow that all follow up, contact and support should also be electronic. In particular, people living with dementia and their carers indicated they preferred telephone and face-to-face contact, rather than just email. Thus, we recommended that researchers should offer participants feedback on study progress and outcomes throughout the study in a choice of formats.

We found that some of the people who acted as ‘testers‘ were unable to tolerate certain devices due to health reasons (e.g. skin irritation) or general discomfort. Conversely, others unexpectedly enjoyed wearing devices despite their initial reservations. Offering prospective participants ‘trial periods’ to wear devices before committing to the study was suggested as one way of helping to minimise early drop out by allaying misgivings and addressing any teething problems in a low pressure way (e.g. swapping wristbands).

Finally, it was also clear to us that carers were deeply involved in the everyday routines of people living with dementia and would be instrumental in maintaining ongoing participation. There was some concern that if not well supported, this could become a burden. As one person wrote:

“*If worn by a dementia patient- who looks after the device - one more job for the carer!!” [*sic*].*

Where individuals have carers and want them to be involved, we would recommend that researchers make provisions to enable this (e.g. scheduling appointments at times when carers can attend). This applies to the process of recruiting participants, following them up over time, providing ongoing technical support and reporting on the findings of studies.

## Discussion

### Acceptability of connected health wearables research

Involving patients and the wider public allowed us to explore the acceptability and feasibility of using wearables as part of future connected health dementia research. The Technology Acceptance Model [14] proposes that user acceptance of technology is affected by two main factors: perceived usefulness, which is “the degree to which a person believes that using a particular system would enhance his or her job performance”; and perceived ease of use, “the degree to which a person believes that using a particular system would be free from effort”. Both of these factors featured among our discussions with potential users. The concept of benefit (or ‘usefulness‘) of wearing a device as part of research was perceived in a variety of ways including increasing insight and self-awareness, motivation to be physically active and personal safety. More broadly, even if individuals had little interest in the device feedback, many saw participating in research for the benefit of society as a sufficiently useful purpose, provided the technology was wearable. Broadly, these findings are consistent with previous research which has reported that older adults do perceive benefits to technology [15] including, specifically, commercially available wearable devices [16].

### Recommendations for conducting future wearables research

In line with the recommendations produced as a result of our involvement activities, the devices we ultimately purchased were low-maintenance, robust and discreet. These deceptively simple requirements meant that we favoured waterproof devices with longer battery life over other, ostensibly more popular, devices that were currently available on the market. We also purchased a greater range of accessories than we originally anticipated in a bid to increase wearability.

One of the most important lessons learned was the need to build in help with set-up and ongoing technical support as part of future connected health dementia research studies, as well as support with operating the device itself. The notion that appropriate infrastructure, investment and support is required to enable older people with long-term conditions to engage with healthcare technology is well-supported in the broader literature [15–18]. For example, support for research involving wearables may share parallels with certain telehealth interventions, which typically involve combinations of sensors and devices (assistive technologies) to remotely measure vital signs and to detect and alert health professionals about emergencies in real time. Whilst it is true that this may increase the financial costs of such research, relying purely on information and support provided by device manufacturers may be a risky alternative. Unlike devices used for telecare however, many commercially available wearable devices have been primarily designed with fitness, rather than healthcare, oriented functions in mind. This could mean that the standard interfaces, settings, default goals (e.g. 10,000 steps per day) and instructions provided by the manufacturer may not be wholly suitable for older research participants, some of whom may be living with multiple long-term health conditions.

Our discussions also revealed queries, caveats and reassurance points regarding how data would be handled and used that needed to be adequately addressed and communicated as part of information given to prospective participants. Previous studies which have explored attitudes towards sharing personal health-related data show that support for health research is contingent on particular conditions being met, such as informed consent and assurances of confidentiality [19–21]. Clearly, when recruiting participants who are living with dementia or cognitive impairments, researchers need to consider how ongoing variation and deterioration in individual capabilities (both on a day to day basis and over the long-term) could affect ability to provide informed consent and ongoing participation. Specific to this field of research was the expectation that any data sharing with commercial companies would be communicated transparently upfront. Indeed, this is important to consider, given that a small minority of the general public find sharing health data with commercial organisations unacceptable, regardless of the potential for public benefit [22],

Furthermore, there was an expectation that researchers should intervene if clear signs were to emerge of treatable health problems requiring medical attention. In telecare, the notion of using digital devices to contribute to diagnostic, monitoring, surveillance and safety purposes is well-established [23, 24]. In connected health dementia research, however, the picture is more complex as data may be collected outside of formal healthcare services and may not necessarily be actively monitored, reviewed or analysed in real-time. Therefore, researchers should give thought to protocols for sharing and acting on data collected and how to communicate these arrangements to prospective participants and their carers.

### Involving people living with MCI, dementia and carers in shaping research

Our work serves as a reminder that people who are living with dementia or cognitive impairments have valuable insights that can enhance the design, delivery and quality of research. Whilst insights gained from involvement activities such as ours are distinct from those gained from research, we believe that they do have value. Indeed, there have been calls for people affected by dementia to have more opportunities to both participate in, and to influence, research [2, 25–27].

Whilst formal ethical approval for involvement (rather than research) activities is not required in England [12], attention to the design of activities and the practicalities were important to enable a variety of people to contribute effectively and on an ethically sound basis. The combination of group discussions, written materials, visual aids and hands-on practical experience with devices took time to develop but was critical to success. In particular, care was taken to offer home testing opportunities without subsequently storing, collecting or analysing the data generated (as would be expected in a research study). This allowed individuals to contribute in different ways and provide relevant insight from having had direct experience of using devices in their everyday lives. Furthermore, attention to the practicalities of workshops such as location, timing of activities, instructions and language was very important in creating the right environment for involvement. Guidance and support from team members and organisations experienced in involving people living with dementia was invaluable in informing our approach [10].

All forms of involvement have their challenges. Although there was no attempt to secure ‘representativeness’, as would be the case in a research study, we aimed to engage a range of people with different diagnoses, needs and experiences. Nonetheless, we experienced some difficulties in engaging certain groups, in particular, those living with MCI. We anticipated that people might not necessarily recognise or identify themselves with this particular label and therefore used the term ‘memory problems’ in our advertisements. Furthermore, we also sought help from colleagues working on a study related to MCI to help advertise our involvement opportunities. These strategies, however, did not overcome this problem. In future, researchers could consider working alongside healthcare professionals and associated services (e.g. memory assessment services) in order to identify people with MCI and others who could be involved in shaping research, such as those with multiple long-term health conditions.

Public involvement can help to identify research questions and priorities that reflect the interests of those affected by dementia. Recently, a James Lind Alliance dementia priority setting partnership involved patients, carers and health and care professionals in identifying and prioritising the top 10 unanswered questions for research [28]. For the involvement activities described in this paper, our primary focus was making timely decisions about the device pool and platform. Nonetheless, we had some preliminary conversations about possible areas for future connected health dementia research, particularly the relationship between sleep and dementia. Better understanding of the potential for connected health dementia research to contribute towards patient and public identified priorities could be a useful and important area to develop in greater depth in future.

## Conclusion

Connected health ‘wearable’ devices present an opportunity to passively and unobtrusively capture high-resolution data from people in everyday life. Coupled with advances in data analysis, these data represent an opportunity to improve the detection, prediction and understanding of diseases like dementia. Whilst still an emerging area of dementia research, we found that in principle, there was good support from patients and the public for this type of research.

By using a range of discussion-based and practical activities, we found it was feasible to involve patients and the public and use their insights to shape the development of a sensing platform for dementia research. Seeking feedback from a range of potential user groups meant that we were better able to attend to their requirements and account for these within development and procurement processes. Furthermore, we identified aspects of research design, setup and support that could potentially support sustained engagement from participants in the future.

## Abbreviations

DPUK: Dementias Platform United Kingdom
MCI: Mild Cognitive Impairment
